# Characterization of extensively drug-resistant *Mycobacterium tuberculosis* isolates circulating in Siberia

**DOI:** 10.1186/1471-2334-14-478

**Published:** 2014-09-03

**Authors:** Maya A Dymova, Andrey G Cherednichenko, Olga I Alkhovik, Eugeny A Khrapov, Tatjana I Petrenko, Maxim L Filipenko

**Affiliations:** Institute of Chemical Biology and Fundamental Medicine (ICBFM), Siberian Branch of The Russian Academy of Sciences (SB RAS), Novosibirsk, Russia; Novosibirsk State University (NSU), Novosibirsk, Russia; Ministry of Public Health and Social Development of The Russian Federation (NRIT), Novosibirsk Research Institute of Tuberculosis, Novosibirsk, Russia

**Keywords:** Mycobacterium tuberculosis, Multidrug resistance, Drug-resistance mutations, Genotyping

## Abstract

**Background:**

The spread of multidrug-resistant (MDR) and extensively drug-resistant (XDR) *Mycobacterium tuberculosis* compromises effective control of tuberculosis (TB) in Siberia. Early identification of drug-resistant isolates is, therefore, crucial for effective treatment of this disease. The aim of this study was to conduct drug susceptibility testing and identify mutations in drug resistance genes in clinical isolates of *M. tuberculosis* from some TB patients presenting for treatment in Siberia.

**Methods:**

Thirty randomly selected clinical isolates of *M. tuberculosis* were obtained from the Novosibirsk Research Institute of Tuberculosis, Russia. Isolates were screened for drug resistance and characterized by variable number of tandem repeats (VNTR)-typing using 15 standard and four additional loci. Deligotyping on multiple large sequences was performed using 10 loci.

**Results:**

Twenty-nine of the isolates were assigned XDR status. Twenty-eight isolates belonged to the *M. tuberculosis* Beijing family, from which 11 isolates were considered the M11 type (39%), two the M2 type (7%), and one the M33 type (3%). Seventeen isolates (60.7%) from this family exhibited unique genetic patterns. The remaining two isolates belonged to the Latino-American Mediterranean family. Gene sequences (*rpoB, katG, rrs, rpsL, tlyA, gidB, gyrA, gyrB*) were analyzed to identify mutations that confer resistance to rifampicin, isoniazid, amikacin, kanamycin, capreomycin, and ofloxacin. The most common mutations among the XDR isolates were S531L in RpoB, S315T in KatG, various codon 94 mutations in *gyrA,* A90V in GyrA, K43R in RpsL, and 1401 A → G in *rrs*; these confer resistance to rifampicin, isoniazid, ofloxacin, streptomycin and kanamycin/capreomycin, respectively. There was high congruence between the two typing methods (VNTR typing and deligotyping) and RD105, RD149, RD152, RD181, and RD207 regions of difference were absent from the 28 Beijing family isolates.

**Conclusions:**

Deligotyping can be used for rapid and reliable screening of *M. tuberculosis* isolates, followed by more in-depth genotyping. Identification of Beijing family isolates with extensive drug resistance confirms that such strains have epidemiological importance in Siberia. Rapid detection of mutations that lead to drug resistance should facilitate selection of effective drug therapies, and the development of early prevention strategies to combat this infection.

**Electronic supplementary material:**

The online version of this article (doi:10.1186/1471-2334-14-478) contains supplementary material, which is available to authorized users.

## Background

The dissemination of extensively drug resistant (XDR) *Mycobacterium tuberculosis* poses a threat to the fight against tuberculosis (TB). According to a 2012 WHO report, XDR-TB has been identified in 84 countries where it comprises 9% of all cases that are multidrug resistant (MDR) [1] from a worldwide figure of 310,000 in 2011. From this, 60% of all MDR-TB cases were from Russia, India, China, and South Africa. In Russia in particular, the number of cases with a confirmed diagnosis of MDR-TB in 2011 was 44,000 [1], while the 2011 Novosibirsk Research Institute of Tuberculosis report for the Novosibirsk region of Russia revealed that the number of cases with primary MDR- and XDR-TB was 272 (28.7%) and 4 (0.4%), respectively. What is particularly alarming is that the rapid increase in MDR-TB in the Novosibirsk region, which in 2005 was 43/100,000, and in 2011 was 58.3/100,000 of the population. In Siberia from 2005 to 2011 the increase was from 34.0 to 46.5/100,000 of the population, while the Far Eastern Federal Districts rose from 20.0/100,000 to 38.3/100,000 of the population.

The causes of XDR TB are multi-factorial and include incorrect treatment regimens, violations of chemotherapy regimens, late diagnosis, and poor infection control [2]. *M. tuberculosis* can rapidly accumulate non-synonymous mutations in genes associated with the emergence of drug resistance. The term XDR itself relates to the involvement of simultaneous resistance to first-line drugs (e.g., isoniazid and rifampicin), one of the injectable second-line drugs (e.g., amikacin, capreomycin, and kanamycin), and any of a number of ofloxacin-based drugs. Rifampicin resistance in *M. tuberculosis* is caused by mutations in the core region of the *rpoB* gene, while isoniazid resistance is associated with mutations in the *katG*, *inhA*, *ahpC,* and *oxyR* genes [3]. Other anti-TB drugs for which resistance has developed include ethambutol (involving mutations in the *embAB* locus), streptomycin (involving nucleotide changes in the *gidB* and *rpsL* genes) [4], ofloxacin (involving the *gyrA* and *gyrB* genes), and amikacin and capreomycin (involving the *rrs* gene encoding 16S rRNA, and also in the *tlyA* gene encoding hemolysin) [5].

The aims of this study were to: 1) genotype and characterize multidrug resistance in *M. tuberculosis* isolates using an extended set of loci for variable number of tandem repeats (VNTR)-typing and by deligotyping; and 2) detect the spectrum and frequency of mutations associated with the emergence of XDR in *M. tuberculosis*.

## Methods

### *M. tuberculosis*isolates

This was a cross-sectional study, examining the drug susceptibility and molecular-genetic features of XDR isolates in Siberia and Far East of Russia. The Novosibirsk Research Institute of Tuberculosis (NRIT) acts as the national referral center for TB in Far East and Siberia region of Russia. Under the National Tuberculosis Control program, randomly selected patients with the MDR and relapse tuberculosis are referred to the NRIT for evaluation and treatment. 372 MDR isolates (31 XDR) were recovered from January 2011 to February 2012. We analyzed 30 consecutive XDR-TB clinical isolates correspond to 1.6% of all the XDR-TB ones isolated in Far East and Siberia region of Russia. All analysed MTB were recovered from individual patients with pulmonary TB who were treated at NRIT from January 2011 to February 2012 were included in study. Despite small size of tested sample set percentage of XDR among MDR isolates (8%) did not showed statistically significant differences (p-value = 0.76) with total one in supervised region (1858 XDR/24400 MDR during the same period of time). All isolates were obtained from patients already receiving TB treatment for more than 1 month. Only three patients were treated for less than 1 month. Fourteen patients were admitted to hospital from the Novosibirsk region, two from the Republic of Khakassia, two from Kemerovo Oblast, two from Omsk Oblast, two from Krasnoyarsk Krai, two from Kamchatka, and one each from Tomsk, Tuva Republic, the Jewish Autonomous Region, Chita, and Altai Republic. This study had bioethical approval from the local committee on medical ethics. Informed consent was received from each patient in the study.

### Bacterial culture and antibiotic susceptibility testing

Drug resistance was determined by calculating the minimal inhibitory concentrations (MIC) using the Bactec MGIT 960 system (BD, New Jersey, USA) with standard concentrations of drugs as follows: isoniazid (0.1 μg/ml), rifampicin (1 μg/ml), pyrazinamide (100 μg/ml), streptomycin (1 μg/ml), ethambutol (5 μg/ml), kanamycin (1 μg/ml), amikacin (1 μg/ml), ethionamide (5 μg/ml), ofloxacin (2 μg/ml), and capreomycin (2.5 μg/ml).

### DNA extraction

DNA was isolated from *M. tuberculosis* cultures at NRIT, as described previously [6].

### PCR amplification and DNA sequencing of genes associated with drug-resistance

PCR amplification was performed in 20-μl volumes containing 65 mM Tris–HCl (pH 8.9), 16 mM (NH4)_2_SO4, 0.05% Tween 20, 2.5 mM MgCl_2_, 0.1 mM dNTPs, 1 μM gene-specific oligonucleotide primers (Table 1), 1 U Taq DNA polymerase, and 1–10 ng of *M. tuberculosis* genomic DNA. The oligonucleotide primer sequences are shown in Table 1. Reactions were carried out in a Tertsik amplifier (DNA Technology, Russia) using an initial denaturation step of 96°C for 3 min, followed by 38 cycles of denaturation at 95°C for 10 s, annealing at 64°C for 10 s, and elongation at 72°C for 20 s, with a final elongation step at 72°C for 3 min. The presence of an amplification product was verified by 6% PAGE with subsequent visualization of the DNA by ethidium bromide staining. PCR products were sequenced in SB RAS Genomics Core Facility according to the manufacturer’s instructions using a Big Dye Terminator v3.1 Cycle Sequencing Kit (Life Technologies Corp., Carlsbad, CA) with the same primers used for PCR and run on an ABI PRISM 3130xl Genetic Analyzer (Life Technologies.). Mutations were detected in the genes by comparison with the *M. tuberculosis* wild-type reference laboratory strain (H37Rv) using Unipro UGENE software (version 1.11.3) (http://ugene.unipro.ru/).

**Table 1:** **Oligonucleotide primers**

Primer	Nucleotide structure
ETRA1	5′-GATTGAGGGGATCGTGATTGG-3′
ETRA2	5′-AAATCGGTCCCATCACCTTCTTAT-3′
ETRB1	5′-GCGAACACCAGGACAGCATCATG-3′
ETRB2	5′-GGCATGCCGGTGATCGAGTGG-3′
ETRC1	5′-GTGAGTCGCTGCAGAACCTGCAG-3′
ETRC2	5′-GGCGTCTTGACCTCCACGAGTG-3′
MIRU02U	5′-CAGGACACGGGTTCTACTG-3′
MIRU02R	5′-GGACTAGGTCGAGGTTGTGTC-3′
MIRU04U	5′-CAGGTCACAACGAGAGGAAGAGC-3′
MIRU04R	5′-GCGGATCGGCCAGCGACTCCTC-3′
MIRU10U	5′-GACTTCCAACAGCACCGTCTTATC-3′
MIRU10R	5′-TCGCACCGATCACGCTACG-3′
MIRU16U	5′-GTTGGAAACGGCGGTTATTGAC-3′
MIRU16R	5′-CGGAGTCGTCCAGCAAGACC-3′
MIRU20U	5′-TCGGAGAGATGCCCTTCGAGTTAG -3′
MIRU20R	5′-TCACGGTCTCCGCACTAACG-3′
MIRU23U	5′-CTCACCAGGATCGCCAAACC-3′
MIRU23R	5′-TCTGACTCATGGTGTCCAACC-3′
MIRU24U	5′-GCTTGTGCGGGAAGGCTA-3′
MIRU24R	5′-CGATCGCGGATCTTTGCT-3′
MIRU26U	5′-CCAGCAGTTGAGCACAGTTCG-3′
MIRU26R	5′-GGATAGGTCCGAGTTCGATTTCC-3′
MIRU27U	5′-CGGTGACCAACGTCAGATTC-3′
MIRU27R	5′-GCGATGTGAGCGTGCCACTCAA-3′
MIRU31U	5′-CTTCGGCGTCGAAGAGAGCCTC-3′
MIRU31R	5′-CGGAACGCTGGTCACCACCTAAG-3′
MIRU39U	5′-CATCGACAAACTGGAGCCAAAC-3′
MIRU39R	5′-GAAACGTCTACGCCCCACAC-3′
MIRU40U	5′-GCAAGAGCAAGAGCACCAAGC-3′
MIRU40R	5′-TGTCTAATCAGGTCTTTCTCTCACGC-3′
MTUB30U	5′-CTTGAAGCCCCGGTCTCATCTGT-3′
MTUB30R	5′-ACTTGAACCCCCACGCCCATTAGTA-3′
MTUB39U	5′-CGGTGGAGGCGATGAACGTCTTC -3′
MTUB39R	5′-TAGAGCGGCACGGGGGAAAGCTTAG-3′
QUB4156U	5′-TGACCACGGATTGCTCTAGT-3′
QUB4156R	5′-GCCGGCGTCCATGTT-3′
V11U	5′- CGCTAGACGTCAGATCCCAG -3′
V11R	5′- GTCTGTTCCGACGCCAATAG -3′
RD105U	5′-GCTGTTTTGCTGTGGATTGTG-3′
RD105R	5′-TCACGTAGCCGCTCAAGAG-3′
RD207U	5′-GAGGATCTTCAGCAGTTGTTCTGG-3′
RD207R	5′-CTTGAAGTACTCGGAAGGTTCG-3′
RD150-1	5′-GTGCTTCGGGGATCAAGG-3′
RD150-2	5′-GGTCTATCCGCTATGTTGTGG-3′
RD181-1	5′-CTGCCGCACAACCAATG-3′
RD181-2	5′-GTGGCGACCAGATCCTTG-3′
RD150-4	5′-GTGCTTCGGGGATCAAGG-3′
RD150-3	5′-GGTCTATCCGCTATGTTGTGG-3′
RD142-U	5′-CGAGTTCAAGGCGATGTTC-3′
RD142-R	5′-GATGTGGTCGTGGTCTCC-3′
RD149-1	5′-GTAGAGGGTTTCCAGTTCCAG-3′
RD149-2	5′- GACGCCGTTGACTTGTTG -3′
RD152-3	5′-GCAGCAACCACCAGGACTC-3′
RD152-4	5′-ACATCAACGCAGCCATCG-3′
RD174-U	5′-CAAGAATCCAAGGCAGAG-3′
RD174-R	5′-CTAACAGCACAAGGTCAC-3′
RD239-1	5′-GACCAACCCTGCTCTTTCTAC-3′
RD239-2	5′-TTGGCGAGGTCTCTTGTC-3′
RD702-U	5′-GGTTGGGTGATGTCGGATTGG-3′
RD702-R	5′-CAGGCGGTCGGTGAATGC-3′
RD711-U	5′-TATCCCGATGACCAATGC-3′
RD711-R	5′-TGCGAGTGATAGATGACG-3′
RD724-U	5′-TCGTAGGGATGACTTGAC-3′
RD724-R	5′-AAGCGTGTAAAGAGGAAC-3′
RD750-1	5′-TCCACGCCAATTTCAAGG-3′
RD750-2	5′-CTCATAGATCACTCGCACAGG-3′

### VNTR-typing

VNTR typing of ETR-A, ETR-B, ETR-C, MIRU 2, MIRU4, MIRU10, MIRU16, MIRU20, MIRU23, MIRU24, MIRU26, MIRU27, MIRU31, MIRU39, MIRU40, Mtub30, Mtub39, QUB4156, and V11 loci was performed by PCR in a volume of 20 μl containing 65 mM Tris–HCl (pH 8.9), 23 mM (NH4)2SO4, 3 mM MgCl2, 0.05% Tween 20, 0.2 mM dNTPs, 1 μM loci-specific oligonucleotide primers, 1 U Taq DNA polymerase and 1–10 ng of *M. tuberculosis* genomic DNA, as described previously [7, 8]. The oligonucleotide primer sequences are shown in Table 1. Reactions were carried out in an iCycler amplifier (Bio-Rad, California, USA) where, after an initial denaturation step of 96°C for 3 min, 38 cycles were performed as follows: denaturation at 95°C for 10 s, annealing at 60°C for 10 s, elongation at 72°C for 30 s, and a final elongation at 72°C for 3 min. The presence of an amplification product was checked by 6% PAGE with subsequent visualization of the DNA by ethidium bromide staining. The number of tandem repeat copies was calculated as a function of the size of the PCR product. The structure and number of repeat copies was also verified selectively by direct sequencing of the amplified DNA fragments. The genotype of each isolate was expressed as a set of 19 digits where each digit showed the number of copies of the corresponding tandem repeat.

### Deligotyping

Deligotyping was performed as described previously [9, 10]. Typing was performed by real-time PCR in a 20 μl volume containing 10 mM Tris-НСl (рН 8.9), 2.5 mM MgCl_2_, 0.2 mM dNTPs, 55 mM KCl, 0.05% Tween 20, 1 U Taq DNA polymerase, and 1–10 ng *M. tuberculosis* genomic DNA. SybrGreen I (supplied as a 10 000 × solution; Life Technologies), was used at a final concentration of 0.2 ×. The oligonucleotide primer sequences are given in Table 1. Reactions were carried out in an iCycler iQ5 (Bio-Rad) with initial denaturation step of 96°C for 3 min, followed by 40 cycles of denaturation at 96°C for 10 s, annealing at 62°C for 20 s, and elongation at 72°C for 10 s, with data acquisition at 72°C on the SYBR channel (excitation at 470 nm; detection at 585 nm using a high pass filter) and melting curves for the amplified products from 82°C to 96°C (in 0.2°C increment).

### Statistical analysis

Statistical data processing was performed using STATISTICA 6.0 (StatSoft, http://www.statsoft.com). Cluster analysis and dendrogram construction were performed using UPGMA and the neighbor-joining method. To determine the congruence of the two methods, the Rand coefficient was used [11]. The cut-off for significance was taken as P < 0.05. The minimum spanning tree (MST) was built using the eBurst algorithm [12]. The Hunter–Gaston Discrimination Index (HGDI) was used as an estimate of the allelic diversity for specific areas in the 19 combined loci [13].

## Results

### Sample description

In our dataset, the patients were aged 20 to 61 (mean 34.9 ± 10.9), with males comprising 58.6%. In Table 2, we introduced artificially age ranges: the minimum value plus one, two and so on standard deviations, obtain several age ranges (20–30,9 (I); 30.9 - 41.8 (II); 41.8 - 52.7 (III); 52,7 – 63, 6 (IV)). Six patients had primary tuberculosis; the others had chronic tuberculosis, where a reinfection or reactivation had occurred. Isolates 133 and 236 were obtained from one patient. Regarding the diagnosis of tuberculosis, patients were divided as follows: fibro-cavernous tuberculosis (17), infiltrative tuberculosis (7), disseminated tuberculosis (3), and caseous pneumonia (2). Only six patients had no comorbidities. Some patients had two or more diseases; for example, hepatitis C was detected among 10 patients (among them one patient had hepatitis B, while two had HIV), bronchitis affected nine patients, different diseases of the gastrointestinal tract affected eight patients, diabetes affected one. Five patients were connected with penitentiary systems, 12 patients had earlier contact with TB patients, of whom eight had family contact. Tuberculosis was diagnosed in 20 patients treated by the general health services, whereas nine cases were obtained from preventive examinations (Table 2).

**Table 2:** **Initial data on the investigated 30 clinical isolates M.tuberculosis observed among 29 tuberculosis patients**

№	Region	S	H	Et	E	Cp	A	K	R	Of	Z	Duration of illness	Age ranges	Sex	DS	Comorbidities	Epidemic history	Complaints
6	2	1	1	1	1	1	1	1	1	1	1	2	I	f	FCPT	No	Family	Cough
9	1	1	1	0	0	1	1	1	1	1	1	2	III	m	FCPT	No	Prison	Cough, shortness of breath
15	3	1	1	1	1	1	1	1	1	1	1	2	II	m	FCPT	HCV, drug addiction	Prison	Cough, shortness of breath, fever,
17	2	1	1	1	1	0	0	1	1	1	1	2	I	f	FCPT	Chronic bronchitis	Family	Cough, hemoptysis
20	4	1	1	1	0	0	1	0	1	0	1	2	II	f	IPT	No	Professional (the nurse)	Cough
21	3	1	1	0	1	1	1	1	1	1	1	1	I	m	IPT	Chronic gastritis	No	Weakness, sweating, fever, hemoptysis
37	3	1	1	1	0	0	1	1	1	1	1	2	II	f	IPT	Neurocirculatory dystonia, encephalopathy, cervical osteochondrosis, cervicalgia, cyst of the right lobe of the liver	Professional (the physiacion)	Shortness of breath
38	9	1	1	1	1	0	0	1	1	1	1	2	I	f	DPT	No	Family	Shortness of breath, weakness
48	10	1	1	1	1	1	1	1	1	1	1	2	II	m	DPT	Peptic ulcer, chronic cholecystitis, HCV, HBV	Family/professional (corpsman in the morgue)	Cough
78	3	1	1	1	1	1	0	0	1	1	1	2	II	f	FCPT	HCV	Family	No
87	3	1	1	0	0	0	0	1	1	1	0	1	I	f	FCPT	Bronchitis after postural drainage	Family	Shortness of breath
94	5	1	1	1	1	1	1	1	1	1	1	2	II	f	FCPT	Obstructive bronchitis, bronchial asthma, syphilis	No	Cough, weakness, shortness of breath
96	4	1	1	1	1	1	0	0	1	1	1	2	I	f	FCPT	HCV	Family	Weaknwss, chest pain
128	6	1	1	1	0	1	1	1	1	1	1	2	I	m	FCPT	Duodenal ulcer	No	Cough
133	7	1	1	1	0	0	1	1	1	1	1	2	II	m	FCPT	Chronic pancreatitis	No	Cough
145	3	1	1	0	1	1	1	1	1	1	0	2	II	m	IPT	HCV, drug addiction	Prison (1998–2001)	Cough
158	3	1	1	1	1	1	1	1	1	1	0	1	I	m	IPT	No	No	Cough, shortness of breath, sweating
169	6	1	1	1	1	0	1	1	1	1	1	2	I	f	FCPT	Respiratory failure 1 level, chronic adnexitis	No	Cough, fever, hemoptysis
171	3	1	1	1	1	0	0	0	1	1	1	2	I	m	FCPT	Secondary bronchial obstruction, respiratory failure 2, heart failure, cachexia	No	Cough, shortness of breath, weakness, chest pain, weight loss
235	3	1	1	1	1	1	0	1	1	1	1	1	IV	m	CP	Diabetes, steatohepatitis, chronic cholecystitis, cholelithiasis	No	Cough, shortness of breath, weakness
236	7	1	1	1	1	1	1	1	1	1	1	2	II	m	FCPT	Chronic pancreatitis	No	Cough
238	3	1	1	1	1	1	1	1	1	1	1	2	IV	m	FCPT	Obstructive bronchitis, emphysema, heart failure	No	Shortness of breath
275	8	1	1	1	1	1	1	1	1	1	1	1	I	f	FCPT	HCV, bronchitis	Contact with neighbors	Cough, weakness, shortness of breath
276	3	1	1	1	1	1	1	1	1	1	0	1	IV	m	DPT	Gastrectomy, HCV, respiratory failure, cachexia	No	Cough, weight less
285	8	1	1	1	1	1	1	1	1	1	1	2	II	m	FCPT	HCV	Prison (2007–2009)	No
287	3	1	1	1	1	1	1	1	1	1	1	2	II	m	IPT	HIV, HCV, sinusitis, stomatitis, drug addiction	No	Cough, shortness of breath, weakness, sweating
300	11	1	1	0	0	1	1	1	1	1	0	2	II	m	FCPT	Obstructive bronchitis	Prison	Cough, fever, chest pain
309	3	1	1	1	1	1	0	1	1	1	1	2	II	m	IPT	HIV, HCV, drug addiction	Contact with neighbors	Cough with sputum
1084	8	1	1	1	1	1	1	1	1	1	1	2	I	m	CP	Obstructive bronchitis	Family	Cough, chest pain
4548	3	1	1	1	1	0	1	1	1	1	1	2	IV	f	FCPT	Obstructive bronchitis, hypertonic disease II	Death	Shortness of breath, tiredness

Note: In the column ‘region’ numbers denote: 1 - Jewish Autonomous Oblast, 2 – the Krasnoyarsk Kray, 3 – Novosibirsk region, 4 – Omsk Oblast, 5 – Chita city, 6 - Kemerovo oblast, 7 – Kamchatka, 8 - The Republic of Khakassia, 9 – The Tuva Republic, 10 - Tomsk, 11 - Altay Republic. Resistance to anti-TB drugs: 1 - resistant, 0 - sensitive. Designation column: S - streptomycin, H - isoniazid, Et - ethionamide, E - ethambutol, CAP - capreomycin, A - amikacin, K-kanamycin, R - rifampicin, Of - ofloxacin, Z - pyrazinamide. In the column “ duration of illness”: 1 - primary tuberculosis, 2 - secondary tuberculosis. In the column «DS» - diagnosis: FCPT - Fibrous-cavernous pulmonary tuberculosis, DPT - disseminated pulmonary tuberculosis, IPT - infiltrative pulmonary tuberculosis, CP - cheesy pneumonia. * - the isolates from one patient.

Only five patients had no complaints at the time of admission to the NRIT (isolates 78 and 285). The other patients complained of cough, shortness of breath, fever, and weight loss. Lesions in the lungs, from single to multiple, and from small to giant size, were detected radiographically. The disintegration of lung-tissues was not detected in one case, but the progression of tuberculosis disease was repeatedly observed in the form of increasing infiltration size (isolate 37). All patients had smear-positive tuberculosis. All cases with identified isolates of the *M. tuberculosis* Latino-American Mediterranean (LAM) family (isolates 15 and 48) were characterized by unfavorable epidemiological histories: one patient was infected in prison, while the second patient worked three years as a nurse in a morgue and was involved in autopsies.

### Drug-susceptibility patterns

Using conventional methods, it was shown that all 30 isolates were multidrug-resistant TB (MDR-TB, i.e. resistant to at least isoniazid and rifampicin); of these, 29 (96,6%) were characterized as extensively drug-resistant, while 10 (33%) were resistant to all of the drugs that were tested.

### Mutations identified in the *rpoB, katG, rrs, tlyA, gidB, rpsL, gyrA, gyrB, and pncA*genes

Mutations in the *rpoB, katG, rrs, tlyA, gidB, rpsL, gyrA, gyrB,* and *pncA* genes were used as markers of the drug resistance phenotypes in the isolates. Sequence analysis of selected isolates of *M. tuberculosis* identified the mutations shown in Table 3.

**Table 3:** **Mutations in** ***rpoB, katG, gidB, pncA, gyrA, gyrB, rpsL, rrs,*** **and** ***tlyA*** **genes associated with the formation of drug resistance and the following amino acid substitutions**

№	rpoB	katG	gidB	pncA	gyr A	gyrB	rpsL	rrs	tlyA
6	S531L, S540R	S315T	E92D	A-11G	S95T	No mutation	K43R	1401 A → G	L11L
9	S531L, S540R	S315T	E92D	I5T	D94A, S95T	No mutation	K43R	1401 A → G, DelA 1473396	L11L
15	S531L, S540R	S315T	L10R	T-7C	D94N, S95T	No mutation	No mutation	1401 A → G, 685 G → A	L11L
17	S531L, S540R	S315T	E92D	48InsG	D94H, S95T	No mutation	K43R	No mutation	L11L
20	L511P, D516G, S540R	S315T	E92D	L4W	S95T	No mutation	No mutation	No mutation	L11L
21	S531L, S540R	S315T	E92D	Y99STOP	A90V, S95T	No mutation	K43R	1401 A → G	L11L
37	S531L, S540R	S315T	E92D	T76P	D94A, S95T	No mutation	K43R	No mutation	L11L
38	S531L, S540R	S315T	E92D	L120P	D94E, S95T	No mutation	K43R	1401 A → G	L11L
48	D516V, S540R	S315T	L10R	T76P	A90V, S95T	No mutation	No mutation	1401 A → G	N/A
78	S531L, S540R	S315T	E92D	A28D	D94G, S95T	No mutation	K43R	No mutation	N/A
87	S531L, S540R	S315T	E92D	D12A	D94G, S95T	No mutation	K43R	No mutation	N/A
94	S531L	S315T	E92D	V139A	S91P, S95T	No mutation	K43R	1401 A → G	N/A
96	H513L	S315T	N/A	No mutation	D94N, S95T	No mutation	No mutation	No mutation	N/A
128	S531L	S315T	E92D	Del G pos256, S179V	N/A	No mutation	K88R	1401 A → G	L11L
133*	S531L	S315T	E92D	N/A	D94G, S95T	No mutation	K43R	1401 A → G	N/A
145	L533P, S540R	S315T	E92D	Del C pos 428	D94G, S95T	No mutation	K43R	No mutation	L11L
158	S531L	S315T	E92D	No mutation	S95T	No mutation	K43R	1401 A → G	N/A
169	S531L, S540R	S315T	E92D	A-11G	D94Y, S95T	No mutation	K43R	No mutation	L11L
171	S531L	S315T	E92D	No mutation	S95T	No mutation	K43R	No mutation	L11L
235	S531L	S315T	E92D	V7G	A90V, S95T	No mutation	K43R	No mutation	L11L
236*	S531L, S540R	S315T	E92D	N/A	D94G, S95T	No mutation	K43R	1401 A → G	L11L
238	S531L	S315T	I81I, E92D	V45G, I31S	D94G, S95T	No mutation	K43R	1401 A → G	L11L
275	S531L	S315T	E92D	No mutation	D94A, S95T	No mutation	K43R	1401 A → G	L11L
276	S531L	S315T	N/A	No mutation	S95T	No mutation	No mutation	1401 A → G	N/A
285	S531L	S315T	E92D	L172P	D94A, S95T	No mutation	K43R	1401 A → G	L11L
287	S531L	S315T	N/A	I31S	S91P, S95T	No mutation	K43R	1401 A → G	N/A
300	S531L, S540R	S315T	E92D, R102P	I31S	A90V, S95T	No mutation	K43R	1401 A → G	N/A
309	S531L, S540R	S315T	N/A	A146T	A90V, S95T	No mutation	K43R	1401 A → G	N/A
1084	S531L, S540R	S315T	N/A	D8N	D94N, S95T	No mutation	K43R	1401 A → G	N/A
4548	S531L	S315T	E92D	Del A pos 353	D94G, S95T	No mutation	K43R	1401 A → G	N/A

Note: *- the isolates from one patient. N/A – we could not get the sequencing.

The presence of a mutation in codon 531 of the *rpoB* gene, which encodes the β-subunit of RNA polymerase and is involved in rifampicin resistance, was found in 27 of the 30 isolates (90%). In particular, among 12 isolates it was the only mutation present in this gene, while the other 14 isolates harbored S531L and S540R mutations. In three cases it was found that isolate 145 contained the mutation in codons L533P and S540R, whereas isolate 48 contained D516V and S540R. In addition, isolate 96 had the H513L substitution in the RpoB protein.

The *katG* gene is involved in the formation of isoniazid resistance. Sequencing of a fragment of this gene revealed the presence of a mutation resulting in a S315T substitution in all the DNA samples from the *M. tuberculosis* isolates (Table 3).

The *rrs* gene, encoding 16S ribosomal RNA, is involved in resistance to amikacin, kanamycin and capreomycin. Among the 30 isolates, 20 had the same nucleotide change at position 1401 (A → G), while a G → A substitution at position 685 was identified in one isolate (no. 15). Additional mutations (Table 3, delA) were found in the intergenic region (between the rrs genes (RVBD 6066) encoding 16S rRNA, and RV6067, encoding 23S rRNA) in the nucleotide position 1473396v (no. 9). Ten isolates did not contain any mutations *in rrs*. The *tlyA* gene, which encodes hemolysin, is also responsible for resistance to amikacin, kanamycin and capreomycin. Analysis of sequenced fragments of this gene showed the presence of synonymous mutations (CTA33CTG, L11L) in several samples (Table 3).

The *gidB* gene, encoding the ribosomal RNA small subunit methyltransferase G, is responsible for resistance to streptomycin. In 23 of the sequenced samples, a mutation (A276C) in *gidB* that results in a nonsynonymous (E92D) change in its protein product was identified; one isolate contained the substitution R102P (no. 300), while another contained a synonymous mutation (I81I) in GidB. Two isolates contained the L10R substitution. In five cases we were unable to amplify the *gidB* gene fragment (Table 3).

In contrast, we found that the *pncA* gene, which encodes pyrazinamidase, exhibited a much higher diversity level in the range of nonsynonymous mutations and sequence deletions, and only five samples did not harbor mutations in this gene (Table 3).

The *gyrA* gene, which codes for the DNA gyrase subunit A, is involved in ofloxacin resistance. In most cases nonsynonymous mutations were found in the 90th, 94th, and 95th codons of this gene. In one case we were unable to amplify this gene (no 128). In the *gyrB* gene, which encodes the DNA gyrase subunit B, no mutations were detected.

The *rpsL* gene, which encodes the ribosomal protein S12, is responsible for streptomycin resistance. In 24 cases (80%) a mutation leading to the amino acid substitution K43R was detected. In five cases no mutations were detected (Table 3).

Taking into account the results of the antibiotic susceptibility testing (Table 2) and genotypic determination of the mutations described above (Table 3), the status of extensively drug-resistance (XDR TB) has been assigned to 29 of the isolates. In addition, isolate 20 had the mutation in the *gyrA* gene that leads to ofloxacin resistance.

### Deligotyping

For deligotyping we used the following loci: RD105, RD149, RD152, RD174, RD181, RD207, RD150, RD239, RD702, RD711, RD724, and RD750, as described by Tsolaki et al. [14]. Oligonucleotide primers were synthesized so that the presence of the amplification product indicated the absence of the deletion in the variable region [10]. Using this technique, we found that 28 of the 30 *M. tuberculosis* clinical isolates did not contain RD105, RD149, RD152, RD181, and RD207 loci in their genomes, thus confirming that they belonged to the Beijing family (Table 4). The presence of RD105, RD181, and RD207 in two of the DNA samples indicated that these isolates belonged to other *M. tuberculosis* families; this was further confirmed by VNTR typing, which showed that these isolates belonged to the LAM family.

**Table 4:** **VNTR–typing and deligotyping results for 30** ***M. tuberculosis*** **isolates**

No	Beijing type	RD-profile	VNTR-profile	VNTR - type
15	-	1,0,0,1,1,1,1,1,1,1,1,1	2,2,2,3,2,1,4,3,2,5,1,6,3,2,5,1,4,10,3	1
48	-	1,0,0,1,1,1,1,1,1,1,1,1	2,2,2,3,2,2,3,3,2,5,1,5,3,2,5,1,4,10,3	2
158	Unknown	0,0,0,1,0,0,1,1,1,1,1,1	3,2,4,3,3,2,3,3,2,5,1,5,3,3,4,4,5,10,6	3
300	Unknown	0,0,0,1,0,0,1,1,1,1,1,1	4,2,4,3,2,2,3,3,2,5,1,5,3,3,3,4,5,10,5	4
309	Unknown	0,0,0,1,0,0,1,1,1,1,1,1	4,2,4,3,2,2,3,3,2,5,1,7,3,3,3,4,5,10,5	5
145	Unknown	0,0,0,1,0,0,1,1,1,1,1,1	4,2,4,3,5,2,3,3,2,5,1,5,2,1,3,4,5,10,5	6
169	Unknown	0,0,0,1,0,0,1,1,1,1,1,1	4,2,4,3,5,2,3,3,2,5,1,5,2,1,3,4,5,10,5	6
235	Unknown	0,0,0,1,0,0,1,1,1,1,1,1	4,2,4,3,5,2,3,3,2,5,1,5,2,1,4,4,5,10,5	7
128	Unknown	0,0,0,1,0,0,1,1,1,1,1,1	4,2,4,3,5,2,3,3,2,5,1,5,2,3,3,4,5,10,5	8
236	Unknown	0,0,0,1,0,0,1,1,1,1,1,1	4,2,4,3,5,2,3,3,2,5,1,5,3,2,3,4,5,10,5	9
94	M2	0,0,0,1,0,0,1,1,1,1,1,1	4,2,4,3,5,2,3,3,2,5,1,5,3,3,3,4,5,10,5	10
133	M2	0,0,0,1,0,0,1,1,1,1,1,1	4,2,4,3,5,2,3,3,2,5,1,5,3,3,3,4,5,10,5	10
20	M33	0,0,0,1,0,0,1,1,1,1,1,1	4,2,4,3,5,2,3,3,2,5,1,6,3,3,3,4,5,10,5	11
87	Unknown	0,0,0,1,0,0,1,1,1,1,1,1	4,2,4,3,5,2,3,3,2,5,1,7,1,1,3,4,5,10,5	12
275	Unknown	0,0,0,1,0,0,1,1,1,1,1,1	4,2,4,3,5,2,3,3,2,5,1,7,2,1,3,4,5,10,5	13
238	Unknown	0,0,0,1,0,0,1,1,1,1,1,1	4,2,4,3,5,2,3,3,2,5,1,7,3,1,3,4,5,10,5	14
285	Unknown	0,0,0,1,0,0,1,1,1,1,1,1	4,2,4,3,5,2,3,3,2,5,1,7,3,1,3,4,5,10,5	14
6	M11	0,0,0,1,0,0,1,1,1,1,1,1	4,2,4,3,5,2,3,3,2,5,1,7,3,3,3,4,5,10,5	15
9	M11	0,0,0,1,0,0,1,1,1,1,1,1	4,2,4,3,5,2,3,3,2,5,1,7,3,3,3,4,5,10,5	15
17	M11	0,0,0,1,0,0,1,1,1,1,1,1	4,2,4,3,5,2,3,3,2,5,1,7,3,3,3,4,5,10,5	15
21	M11	0,0,0,1,0,0,1,1,1,1,1,1	4,2,4,3,5,2,3,3,2,5,1,7,3,3,3,4,5,10,5	15
37	M11	0,0,0,1,0,0,1,1,1,1,1,1	4,2,4,3,5,2,3,3,2,5,1,7,3,3,3,4,5,10,5	15
38	M11	0,0,0,1,0,0,1,1,1,1,1,1	4,2,4,3,5,2,3,3,2,5,1,7,3,3,3,4,5,10,5	15
78	M11	0,0,0,1,0,0,1,1,1,1,1,1	4,2,4,3,5,2,3,3,2,5,1,7,3,3,3,4,5,10,5	15
276	M11	0,0,0,1,0,0,1,1,1,1,1,1	4,2,4,3,5,2,3,3,2,5,1,7,3,3,3,4,5,10,5	15
287	M11	0,0,0,1,0,0,1,1,1,1,1,1	4,2,4,3,5,2,3,3,2,5,1,7,3,3,3,4,5,10,5	15
4548	M11	0,0,0,1,0,0,1,1,1,1,1,1	4,2,4,3,5,2,3,3,2,5,1,7,3,3,3,4,5,10,5	15
96	Unknown	0,0,0,1,0,0,1,1,1,1,1,1	4,2,4,3,6,2,2,3,2,5,1,3,3,3,3,4,5,10,5	16
171	Unknown	0,0,0,1,0,0,1,1,1,1,1,1	4,2,4,3,6,2,3,3,2,5,1,7,3,2,3,4,5,10,5	17
1084	M11	0,0,0,1,0,0,1,1,1,1,1,1	6,2,4,3,5,2,3,3,2,5,1,7,3,3,3,4,5,10,5	18

Note: Each number in the column “RD-profile” corresponds to the presence (1) or absence (0) of the region of differences (RD105, RD149, RD152, RD174, RD181, RD207, RD150, RD239, RD702, RD711, RD724, RD750) in the corresponding part of the genomic DNA sample. Similarly, in the column “VNTR-profile” each digit represents the number of repeats at a locus: ETRA, ETRB, ETRC, ETRD, ETRE, miru02, miru10, miru16, miru20, miru23, miru24, miru26, miru27, miru39, miru40, Mtub30, Mtub39, Qub4156, and V11.

RD149 and RD152 loci were absent in all the 30 mycobacterial DNA samples analyzed. However, RD150, RD239, RD702, RD711 and RD724 loci were found in all of the DNA samples The Hunter–Gaston discrimination index (HGDI) for deligotyping was equal to 0.128.

### VNTR-typing

*M. tuberculosis* genetic families were determined using VNTR typing and the MIRU–VNTR*plus* database (http://www.miru-vntrplus.org/MIRU/index.faces) [15]. We hypothesized that most of the *M. tuberculosis* isolates characterized as having extensive drug resistance would belong to the Beijing family. Hence, we used the 15 standard VNTR-loci proposed by Supply et al. for genotyping [16]. For enhanced differentiation we used four additional loci (Mtub30, Mtub39, Qub4156, V11) for closely related isolates of the Beijing family, which proved to be highly discriminatory [17]. It was shown that among the 30 clinical isolates, 28 belonged to the Beijing family (Table 4). Using the classification of Mokrousov [18], we identified 28 isolate types of the Beijing family: 11 isolates (39.3%) belonged to the M11 type, 2 (7.1%) were the M2 type, while one isolate (3.6%) was the M33 type. The remaining 14 isolates (50.0%) could not be attributed to any known type based on this classification method; hence, they have been labeled ‘unknown’. For each locus, the HGDI and its associated confidence intervals was calculated (Table 5). Additionally, the total HGDI for VNTR-typing was calculated as 0.889.

**Table 5:** **Hunter-Gaston diversity indeх with confidence intervals for each locus**

Locus	Diversity index	Confidence interval	K	Max (pi)
MIRU26	0.609	0.550 - 0.667	5	0.548
MIRU39	0.574	0.501 - 0.648	4	0.613
MIRU31	0.441	0.343 - 0.539	5	0.742
MIRU27	0.385	0.291 - 0.479	4	0.774
ETRA	0.299	0.199 - 0.399	5	0.839
MIRU40	0.297	0.198 - 0.395	4	0.839
V11	0.243	0.148 - 0.338	4	0.871
MIRU10	0.187	0.099 - 0.275	4	0.903
MTUB30	0.185	0.099 - 0.271	3	0.903
ETRC	0.185	0.099 - 0.271	3	0.903
MTUB39	0.185	0.099 - 0.271	3	0.903
MIRU2	0.127	0.051 - 0.203	3	0.935
MIRU16	0.065	0.008 - 0.121	2	0.968
MIRU20	0.065	0.008 - 0.121	2	0.968
MIRU24	0.065	0.008 - 0.121	2	0.968
MIRU23	0.065	0.008 - 0.121	2	0.968
ETRD	0.065	0.008 - 0.121	2	0.968
QUB4156	0.065	0.008 - 0.121	2	0.968
ETRB	0.065	0.008 - 0.121	2	0.968

### Cluster analysis by VNTR

**Figure 1:**
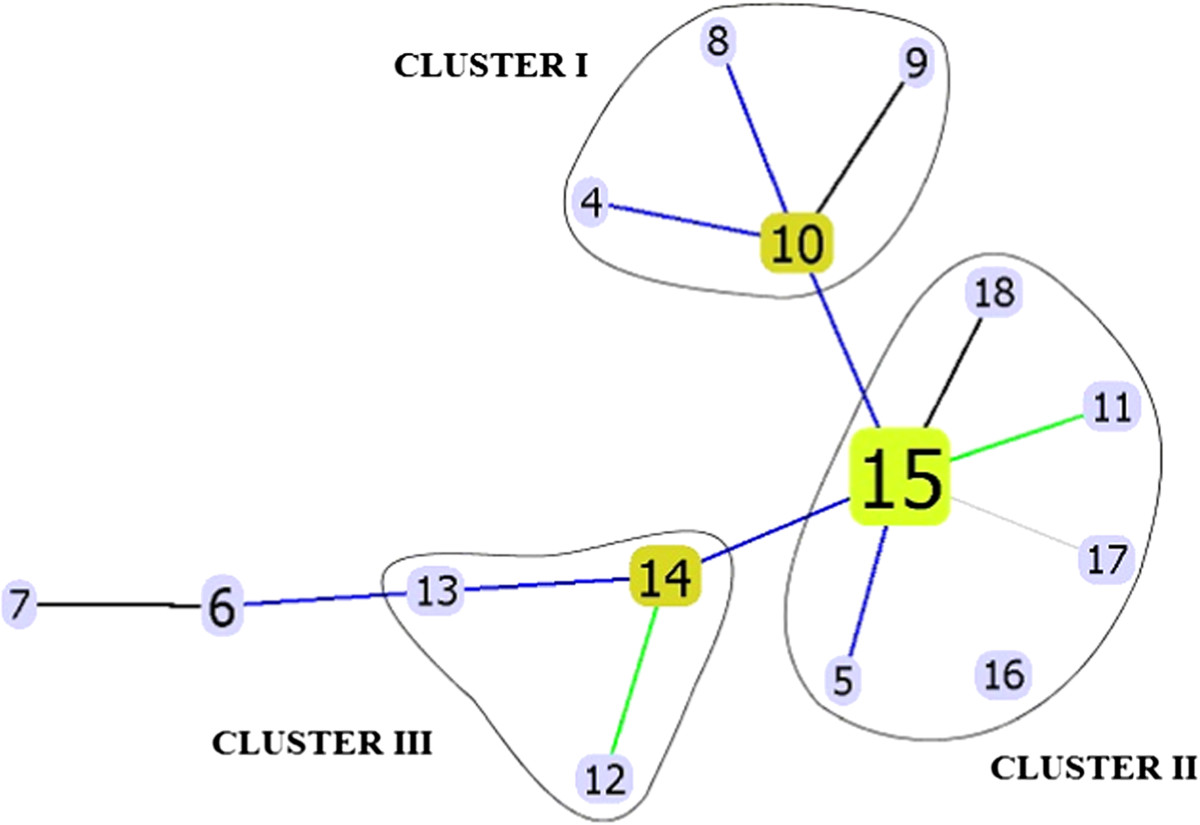
**Minimum spanning tree based on VNTR-typing of 30** ***M .tuberculosis*** **isolates using 19 polymorphic loci.** Note: There are solid line circled clusters isolates (I, II, III) with similar the genetic profile.

To elucidate the phylogenetic relationships between the *M. tuberculosis* isolates we constructed a MST based on VNTR-sections (Figure 1). This tree is a branched chain wherein each vertex (a square with rounded corners) represents a VNTR-type (Table 4), and the square is specified by the number of types. Vertex size is directly proportional to the number of *M. tuberculosis* isolates with relevant VNTR-profiles. The tops of the tree branches are connected by edges, where the color denotes the difference between the two VNTR-types: black and blue represents a difference in one locus, green and dark gray represent differences in two different loci, and light gray represents differences in three loci. On the minimum spanning tree, we identified three clusters containing from three to six VNTR-types on the inside; these isolates differ from each other in a single copy at the locus analyzed. Three VNTR-types (nos. 1, 2, and 3, Table 4) had significantly different genetic profiles, and were not, therefore, included in the MST. VNTR-types 1 and 2 are consistent with *M. tuberculosis* DNA samples 15 and 48 that belong to the LAM family. VNTR-type 3 corresponds to sample 158, and is referred to by us as ‘Beijing family group unknown’. Cluster I comprised the VNTR-types 4, 8, 9, and 10, wherein the latter corresponds to the M2 type. Cluster II contains six VNTR-types (5, 11, 15, 16, 17, and 18): the most numerous of these (no. 15), consisted of 10 isolates of the M11 Beijing *M. tuberculosis* family. According to the scientific literature, the M2 type is dominant in Russia, but is also found in East Asia, albeit at a lower frequency [19]. Cluster III consists of three VNTR-types (nos. 12, 13, and 14), which are connected to two other types (nos. 6, and 7) at the level of one or two VNTR-loci changes, respectively. Although these VNTR-types (nos. 6, 7, 12, 13, and 14), as based on their characteristic VNTR-profiles, belong to the Beijing family, they did not have RD207 and RD105 regions of difference so we were unable to relate them to any of the known classification types described by Mokrousov [18].

## Discussion

In this work, 30 isolates *M. tuberculosis* from 29 patients with TB were typed. One patient had a mixed population of *M. tuberculosis* genotypes. According to the scientific literature, in regions with high rates of TB morbidity and mortality, or in HIV-infected patients with tuberculosis, mixed populations of *M. tuberculosis* in humans might exist [20].

In this study, we used a traditional method, VNTR-typing, with 15 standard loci and 4 highly discriminatory loci for closely related strains, to analyze our isolates. Loci with the most discriminatory power were MIRU26, MIRU39, and MIRU31. What is unusual is that V11, Mtub30, Mtub39 and QUB4156 loci displayed relatively low Hunter–Gaston discrimination indices; this could be related to the distribution of certain genotypes in the Siberian and Far Eastern Federal Districts. The statistical analysis showed that the Rand index for the two typing methods was equal to 1 (i.e., the results of the deligotyping and VNTR-typing were consistent with each other). It should be noted that HGDI for the VNTR-typing was higher (HGDI = 0.889) than the deligotyping (HGDI = 0.128) [13]. This may be caused by an enhanced rate of mutation in the variable tandem loci compared to regions of difference [21]. Despite the low HGDI, deligotyping is a quick and convenient method for molecular-epidemiological prescreening of *M. tuberculosis* samples, prior to more in-depth typing of mycobacteria by other methods.

Thus, in this study, among 30 *M. tuberculosis* isolates, of which 29 were characterized as XDR, 93.3% belonged to Beijing family. We have previously shown that among isolates from patients with complicated forms of progressive pulmonary tuberculosis there is a predominance of isolates of this family (i.e., 84% (37 of 44) [22]. In the present study, the frequency of Beijing family isolates from patients with severe forms of pulmonary tuberculosis in the city of Novosibirsk is significantly different from that in the general population (OR 7.1, 95%CI [2.93–17.53], P < 0.0001). Our findings are consistent with those reported in other countries; for example, in Nepal, genotyping of XDR-TB isolates showed an overall prevalence of the Beijing family of 69% [23]. Taken together, the above data confirm that the Beijing family of *M. tuberculosis* is highly virulent in humans.

In the resulting minimal spanning tree, the VNTR-type 15, which corresponds to the M11 type, had the largest size, occupied a central position, which once again confirms its position in the ancestral population structure of the Beijing family [18]. We found that the distribution of the M11 type in our dataset was 36%, while the M2 type was 6%. Previously, several dominant isolate types of the Beijing family were identified that have epidemiological significance in the Russian Federation [24]. Five types are reported to occur at the greatest frequency, namely, M2, M8, M11, M28, and M33 [19]. M11 is the ancestral line that dominates Eurasia but is not found in Australia and South Africa. Isolates of this type have high adaptability and the ability to increase in numbers in THP-1 macrophages, as well as a high capacity to induce synthesis of the anti-inflammatory cytokine IL-10, and to induce macrophage necrosis. These biological characteristics in mycobacteria contribute to increased transmissibility and virulence, so identification of such strains using molecular genetic techniques will contribute to more effective use of drug therapy, and inform decisions on standard molecular epidemiological-related problems in TB. Identifying two isolates belonging to the LAM family is not surprising. This family is widespread and was found for the first time in Latin America, followed by the Mediterranean Basin [25]. In Russia, this family has the second highest incidence in the mycobacterial families after the Beijing type; these two families (LAM and Beijing) are associated with resistance to rifampicin and streptomycin [26]. Our spanning tree analysis showed that despite identical VNTR-profiles, isolates belonging to one cluster had different mutations in genes associated with the formation of drug resistance. For example, nine isolates belonging to MST No. 15 (Figure 1) have different mutations in *pncA* and *gyrA* genes, primarily in pncA.

Another aspect of our work was focused on analyzing drug resistance in the *M. tuberculosis* clinical isolates. The scientific literature refers to this type of isolate as pre-XDR-TB, or pre-totally drug-resistant-(TDR)-TB because the formation of resistance to additional drugs is a result of mutational events whereby such bacteria have become XDR and TDR, respectively. We searched for mutations in the genomes of the *M. tuberculosis* isolates that were associated with the formation of drug resistance using molecular genetics methods. It is known that the presence of non-synonymous mutations in the *rrs* gene causes resistance to streptomycin, amikacin, kanamycin, and capreomycin, whereas such substitutions in *tlyA* only confer resistance to amikacin, capreomycin and kanamycin. Resistance to streptomycin is conferred by non-synonymous changes in *gidB and rpsL*, while ofloxacin resistance is conferred by such changes in *gyrA* and *gyrB*. Finally, non-synonymous substitutions in *pncA, rpoB,* and *katG* confer resistance to pyrazinamide, rifampicin, and isoniazid, respectively.

In Siberia, a nucleotide substitution at codon 531 (S531L) in *rpoB* is dominant in rifampicin-resistant isolates. Mutations in other codons of this gene are less common, but are associated with lower relative fitness in isolates carrying this mutation compared with those carrying the wild-type gene [27, 28]. In addition, it is worth noting that there is a relationship between the minimum inhibitory concentration of rifampicin and the presence of a mutation in the core region of *rpoB*. It was found that a high degree of resistance to rifampicin (MIC ≥ 50 μg/ml) was observed in isolates which have mutations in codons 513, 522, and 531. In contrast, a low degree of drug-resistance (MIC ≥ 12.5 μg/ml) is conferred by nucleotide substitutions in codons 514, 521, 533 [29]. Mutations in codon 516 are also associated with a high degree of resistance (MIC ≥ 64 μg/ml) [30, 31]. Although some studies report similar results, H526L and H526N mutations are the exception; in these cases, the MIC values did not exceed 8 and 16 μg/ml, respectively. A replacement mutation (L533P) was associated with MIC > 32 μg/ml. Interestingly, Zhou et al. showed that replacement mutations in codons 526, 531, 532, 533 and 563 of *rpoB* in *E.coli* destabilize the promoter initiation complex which correspond to a range of transcription activity at these promoters at different growth rates [32].

The most common mutation for isoniazid resistance in the XDR strains was at codon 315 of the *katG* gene, thus correlating with the data from other studies [33]. This mutation was found among all of the isolates analyzed. Isoniazid resistance can also be caused by changes in the *ahpC*, *inhA*, *kasA* and *oxyR* genes and it may be necessary to study these genes to investigate the molecular basis for isoniazid resistance in our XDR *M. tuberculosis* isolates [34].

The dominant mutations in the *rpsL* gene, which is involved in resistance to streptomycin, lead to amino acid substitutions in codons K43R and K88R. It is worth noting that the mutation at codon 43 (K → R) is strongly associated with high-level resistance to streptomycin, which worsens the prognosis for patients undergoing treatment for infection with *M. tuberculosis* carrying this mutation.

Resistance to the aminoglycosides amikacin and kanamycin and to capreomycin is thought to be associated with mutations in the 16S rRNA gene (*rrs*). Mutations in rRNA 16S (*rrs*) at position 1401 are associated with the development of high resistance to these drugs, and this mutation was common in our dataset (66%). The *tlyA* gene, coding for cytotoxin/hemolysin, is involved in resistance to aminoglycosides and capreomycin as well, but analysis of this gene was not informative because only 10 isolates had synonymous mutations (L11L).

The most common mutations for ofloxacin resistance were in the *gyrA* gene at codons 94 and 90, the former of which had the highest frequency of mutation (33%). This is consistent with reports on XDR strains from Pakistan, India, China, the United States, and Germany [35–38], although in other regions different distributions of mutations may occur [39]. While the sequence change at codon 94 exhibited the highest variability, 63.3% of the XDR strains had a mutation at codon 95 of the *gyrA* gene; however, this mutation does not contribute to ofloxacin resistance and instead serves as an evolutionary marker for classification of *M. tuberculosis* strains into three principal genetic groups (PGGs) [40]. Hence, the ACC polymorphism at codon 95 confirms the PGG1 and PGG2 grouping [41]. In contrast, we found no mutations in *gyrB*.

The *pncA* gene, which encodes pyrazinamidase, hydrolyzes pyrazinamide to pyrazinoic acid, the latter in turn communicates with the 30S ribosomal protein S1 (RpsA), thus the protein cannot bind to tmRNA molecules that inhibit trans translation in mycobacteria [42]. It remains unclear why there is a variety of mutations in the absence of any common mutations in this gene. One hypothesis that may explain the wide variety of nucleotide substitutions is that because its protein product is small (186 amino acids), each mutation may adversely affect its function [3]. This requires further study.

Thus, sequence analysis of the hot spot regions of various genetic loci revealed that the most common mutations among the XDR isolates were S531L of RpoB, S315T of KatG, various mutations in codon 94 of *gyrA* and the A90V substitution in the GyrA protein, K43R of RpsL, and 1401 A → G of *rrs* for rifampicin, isoniazid, ofloxacin, streptomycin and kanamycin/capreomycin resistance, respectively. It is likely that these major mutations do not have a large effect on bacterial fitness [28]. Other studies have also reported similar mutations among XDR-TB isolates from different countries. It is possible that the disagreement between the genotypic and phenotypic resistance to ofloxacin and amikacin/kanamycin/capreomycin we observed may be caused by other mutations in regions distinct from the currently known target regions in these drugs [43]. In addition, mycobacterial cultures may also comprise a mixed population of resistant and sensitive bacteria [44] and, therefore, DNA extracted from specimens may contain lower levels of the resistant genotype, leading to a false low detection rate for the specimen. It should be noted that there are also mutations that are responsible for the formation of low level resistance to particular drugs. Nevertheless, sequencing the regions of genes responsible for the development of resistance to anti-TB drugs can be a convenient way to quickly identify drug resistance, thus enabling selection of effective drugs for treatment of the disease.

## Conclusions

Identification of Beijing family *M. tuberculosis* isolates that are extensively drug-resistant and have high transmissibility and virulence highlights the need for early and rapid detection methods for this pathogen. Such isolates can be characterized using VNTR-typing and deligotyping methods. Deligotyping is a simple and convenient method for pre-screening large numbers of samples in epidemiological research, but the method has low discriminatory power. Evaluation of genetic diversity in *M. tuberculosis* strains circulating within an area will enable authorities to adopt effective measures to prevent the spread of infection, as well as providing basic information about the relationship between genetic diversity and virulence and immunity in the host. Armed with such information, it may be possible to determine how genetic diversity contributes to the global distribution of MDR and XDR in *M. tuberculosis*. It is obvious that there are epistatic interactions between the different mutations associated with the emergence of drug resistance, the genetic environment, and compensatory mutations, but understanding these complex relationships will require further research. DNA sequencing has revealed the presence of major mutations in the genes responsible for the emergence of resistance to the major anti-TB drugs. It is hoped that in the future, identification of *M. tuberculosis* drug resistant isolates and a description of their phenotypic properties will inform selection of more effective drugs against TB in the complex epidemiological situations that exist in Western and Eastern Siberia.

### Ethical approval

Ethical approval was obtained from the Bioethical Committee of Institute of Chemical Biology and Fundamental Medicine Siberian Academy of Science. Informed consent was obtained from each patient in the study.

## Authors’ original submitted files for images

Below are the links to the authors’ original submitted files for images. Authors’ original file for figure 1
