# Supplementary figures and images for: Characterization of extensively drug-resistant Mycobacterium tuberculosis isolates circulating in Siberia
Source: BMC Infect Dis. 2014 Sep 3;14:478. doi: 10.1186/1471-2334-14-478 (PMC4161839; doi:10.1186/1471-2334-14-478)

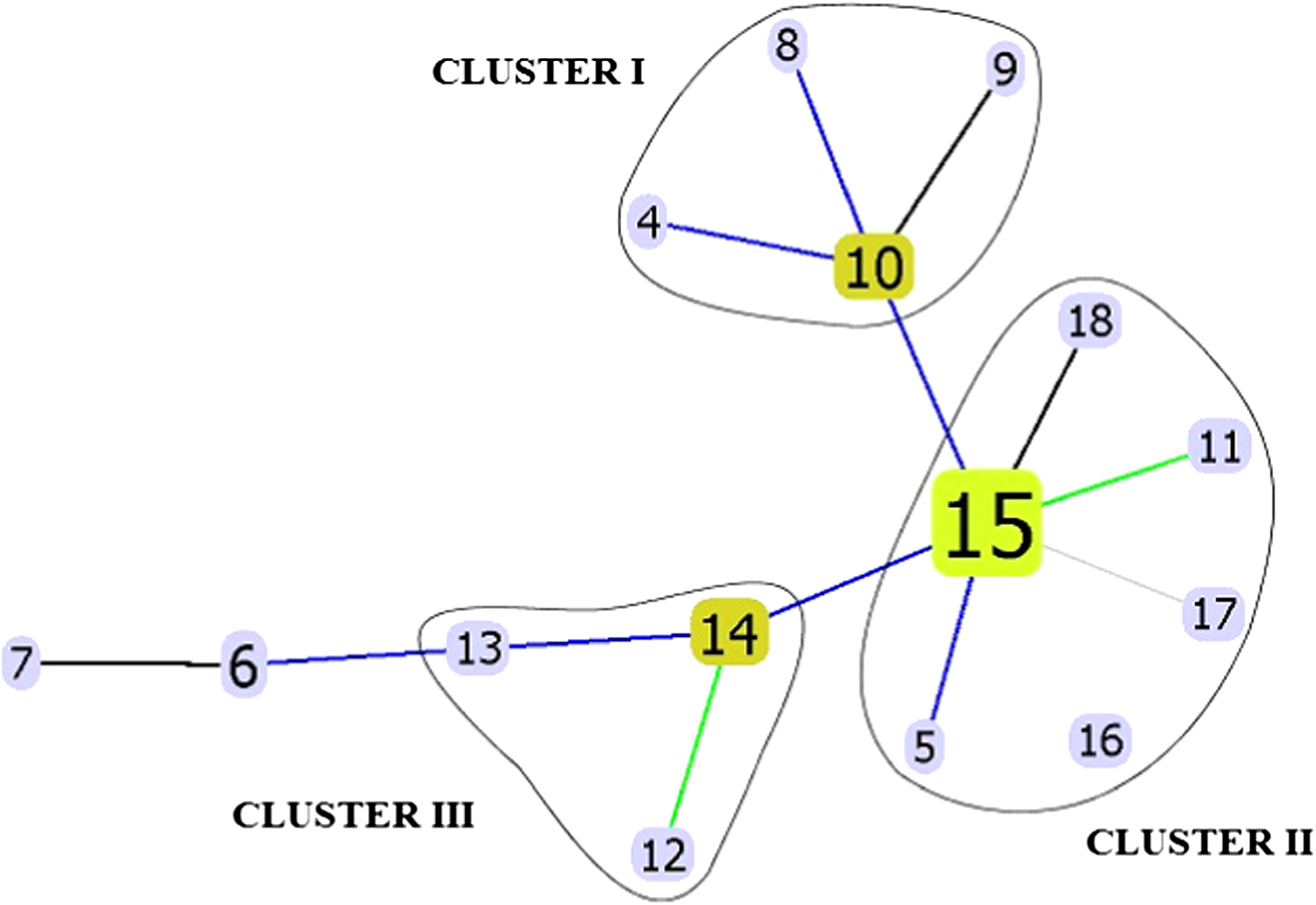

Supplement: Supplementary file 1 — Authors’ original file for figure 1 [file 12879_2013_3783_MOESM1_ESM.tif]
